# Revisiting Pseudo-Haptics for Psychomotor Skills Development in Online Teaching

**DOI:** 10.7759/cureus.23664

**Published:** 2022-03-30

**Authors:** Bill Kapralos, Alvaro Quevedo, Celina Da Silva, Eva Peisachovich, KC Collins, Kamen Kanev, Adam Dubrowski

**Affiliations:** 1 Software Informatics Research Centre, Ontario Tech University, Oshawa, CAN; 2 Faculty of Business and Information Technology, Ontario Tech University, Oshawa, CAN; 3 Medical Education and Simulation, York University, Toronto, CAN; 4 School of Information Technology, Carleton University, Ottawa, CAN; 5 Research Institute of Electronics, Shizuoka University, Hamamatsu, JPN; 6 Research and Development, Ontario Tech University, Oshawa, CAN

**Keywords:** virtual reality, pseudo-haptics, psychomotor skills, remote learning, virtual learning environment

## Abstract

In a centralized model of simulation-based education (Ce-SBE), the trainees practice clinical skills in simulated laboratories based on physical models, while in a decentralized model (De-SBE), the trainees practice these skills outside of these laboratories. Attention to De-SBE has drastically shifted to virtual learning environments (VLEs), serious games, and virtual simulations employing various digital technologies, including virtual, augmented, and mixed reality. In particular, remote learning has grown immensely during the COVID-19 pandemic as traditional in-person teaching and training activities are conducted online as a form of facilitating continuity in education. VLEs allow trainees to learn from virtual simulated health experiences in an interactive, engaging, and ethically safe manner, while providing educators the opportunity to implement simulated experiences to a larger number of learners. Despite these benefits, for certain types of clinical skills, such as psychomotor skills, VLEs have not yet reached their potential. This is primarily due to technical limitations and cost issues with the haptic devices required to simulate the sense of touch. Pseudo-haptic refers to the illusion of haptic stimulation in the absence of mechanical haptic interfaces and often combines the use of a passive input device (e.g., mouse) with visual and auditory feedback to simulate haptic properties (stiffness or friction of an object). Although the application of pseudo-haptics for psychomotor skills development is still in its infancy and currently trending due to the availability of consumer-level technologies, the potential to present haptic cues in the absence of active haptic devices may allow trainees to practice some tasks outside of research and training labs. The implications of pseudo-haptics are tremendous, particularly as remote learning becomes more widespread, and warrant further discussion.

## Editorial

Simulation provides a viable and safe alternative to real-world practice, allowing learners to train until they achieve a speciﬁc competency level [1]. The traditional simulation-based education model can be described as a centralized model of simulation-based education (Ce-SBE), whereby the learners congregate in simulation laboratories to practice a range of skills across many learning domains. Although simulation-based training has been widely adopted as a part of health professions education, the required infrastructure, maintenance, and personnel can be prohibitive [2]. Immersive virtual learning environments (iVLEs), including virtual simulations and serious games, that is, video games applied specifically to learning and training, have been developed to provide effective educational tools for training across a wide variety of fields, in an engaging, safe, and cost-effective manner [3]. Current advances in immersive technologies are providing affordable consumer-level devices that allow for the recreation of real-world experiences for trainees to safely and engage in interactive and immersive activities. The popularity and application of iVLEs has witnessed exponential growth recently, as we moved to remote learning due to lockdowns/stay-at-home orders because of coronavirus disease 2019 (COVID-19). Such a decentralized model (De-SBE), where the trainees practice clinical skills outside of simulation laboratories has been facilitated by advancements in technology, specifically immersive technologies.

However, the majority of iVLEs are still focused on cognitive and affective skill development; that is, development of the mental skills and the acquisition of knowledge, as a result of the technology being driven mostly by visual and auditory feedback commonly available in consumer-level devices such as laptops, desktop computers, and mobile devices. Currently, accurately simulating the sense of touch (inherent in psychomotor skills development) is difficult as it requires providing a realistic perception of somatosensory cues including pressure, temperature, position, movement, texture, and vibration through actuators embedded in high-end haptic devices [4]. While there are several high-end readily available haptic devices, the lack of consumer-level technological advancements in haptics has created an entry-level barrier for iVLEs to be extensively used in De-SBE to develop psychomotor clinical skills where trainees typically have access to basic computing equipment such as a mouse and keyboard. Furthermore, the cost of a haptic device is proportional to its haptic fidelity (i.e., higher fidelity implies higher realism and therefore a higher cost). Given the lack of technological advancements and associated high costs of haptic devices , iVLEs have not been used extensively in De-SBE to develop psychomotor clinical skills, which include for instance suturing, intravenous (IV) placements, intraosseous (IO) placements, tissue preparations, catheterization, needle and syringe injections, superficial and deep wound care among other clinical skills. The thesis of this editorial is that iVLEs can still be used in a De-SBE model of training psychomotor clinical skills, when complex and costly haptic devices are replaced with more common devices to form a percept termed “pseudo-haptics”. The use of pseudo-haptics in iVLEs may enable application in a De-SBE model of training for some psychomotor clinical skills.

The case for pseudo-haptics 

The illusion of haptic sensations without the use of active haptic interfaces is a powerful one [5]. For example, the classic rubber hand illusion including two participants, has a rubber hand placed alongside the real hand of Participant 1, while their real hand is covered from the view. Then Participant 2 strokes both the rubber hand and real hand at the same time. The visual decoupling causes Participant 1 to experience the rubber hand as their own to the point where, when Participant 2 injects a needle in the rubber hand, Participant 1 interprets this as having been done to the real hand and feels the sensation of the needle [5]. Pseudo-haptics is grounded in the work of Aldridge et al. [6] who observed that the visual representation of a virtual object can affect the integration of the touch-based feedback. Lécuyer et al. [7] developed a novel interaction technique that simulates textures in desktop applications by using a passive input device (mouse) combined with visual feedback. Their technique relies on the control/display (C/D) ratio, that is, the ratio between the amplitude of movements of the participant’s real hand and the amplitude of movements of the virtual cursor [7], and consists of modifying the motion of the cursor when it passes over simulated textures. The C/D ratio is adjusted as a function of the simulated “height” of the terrain the mouse cursor is traveling. Experimental results showed that participants were successfully able to identify bumps and holes by only using the variations of the cursor motion [7]. More recently, Li et al. [8] developed a surgical training system that employed pseudo-haptics to identify tumors upon palpation, in which the firmness of the tumor was presented using pseudo-haptics. Although a complete review of pseudo-haptics is beyond the scope of this article, Ujitoko and Ban [9] provide a recent and thorough review of the design and application of pseudo-haptics, focusing on three categories of use (training, assistance, and entertainment). They observed that the number of pseudo-haptics applications for training are far less than those applied to entertainment and assistance (e.g., navigation on touch-screen interfaces, and its use to influence the memorization of important knowledge), or entertainment [9]. This begs the question, can pseudo-haptics provide a viable alternative to complex expensive haptic devices required to facilitate psychomotor skill development in the virtual domain? Spurred on by the COVID-19 pandemic and resulting healthcare training laboratories, it is apparent that there is a rise with difficulties to support hands-on (psychomotor) skill training in a remote setting. Hence, we have begun investigating the application of pseudo-haptics for healthcare professions training applications. We primarily focused on virtual learning environments (virtual simulations and serious games) in a remote (online) setting.

Pseudo-haptics and psychomotor skills development

For illustrative purposes, this section will present some examples of potential applications of pseudo-haptics applied to medical training. First, we will discuss pseudo-haptics in bone-drilling, outlining some of our current preliminary work in this domain. We then propose several other common medical procedures that we believe show promise for pseudo-haptics which we will investigate as part of future work.

Bone-drilling is a psychomotor orthopedic skill that relies on the use of cross-sensory feedback including vision, sound and haptics. Drilling is a task that requires training to master the multimodal sensory feedback and used in various medical procedures including dental bone and implant surgery [10], orthopedic surgery [11], and needle insertion [12], amongst others. Such procedures require attention beyond just vision since sound and touch can play an important role when vision is obscured. We conducted experimental pilot work whereby 13 participants were asked to complete a virtual drilling task (drilling a block of virtual wood) under a combination of visual, auditory, and kinesthetic (mouse movement using a standard computer mouse or track pad) conditions [13]. The experiment was conducted remotely, facilitated by Google Meet. We hypothesized that although each participant’s availability of a haptic device in their home could not be guaranteed, it was safe to assume that every participant had access to a computer mouse. Therefore, fundamental haptic cues associated with grasping the mouse using a hand and moving the mouse back-and-forth, along with auditory and visual feedback (in various combinations) can simulate a drilling task. The results from this experiment confirmed our hypothesis; using auditory feedback in conjunction with kinesthetic feedback obtained with a standard computer mouse allowed participants to complete the required drilling task accurately.

Computer-based simulation for nursing procedure training has gained relevance, as haptic interaction devices can develop fine motor skills, including needle insertion. Needle insertion includes many factors such as the needle size and length, the body region and tissue it is inserted into, and assessments involved prior to inserting the needle, such as light palpation and landmarking, which may pose challenges when using computer simulation. Other advanced nursing skills include removing epicardial pacing wires (i.e. freeing a pacing wire from the skin and applying gentle traction). Chest tube removal is another advanced competency that would benefit from removing the sutures, clamping, and pulling the tube. Other potential haptic experiences include accessing port-a-caths for patients requiring frequent and long-term oncology therapy, care of acute and chronic wounds such as amputations and burns; as well, insertions of nasogastric tubes and urinary catheters.

It is also important to consider communication and healing touch essential to communication yet absent in virtual human interactions. Using pseudo-haptic methods can support non-verbal communication through touch, hence enhancing human interaction. This sense of touch can become revolutionary when conducting simulated person interactions to provide bidirectional touch to support teaching healing touch, including verbal communication and nonverbal communication skills. Within the context of a simulated nurse-patient interaction, investigating how touch can be used to communicate is essential to our evolution as human beings simulating human-to-human touch or virtual human-to-human touch. The ability to support this idea can further explore the need for touch as a human experience by being combined with speech, social interactions, and empathy building. Finding ways to evaluate haptic interaction perception objectively represents a promising research field and application of pseudo-haptics.

Summary

Although the cost of immersive technologies continues to decrease, most iVLEs (virtual simulations and serious games in particular), are still focused on cognitive skill development, that is, the development of mental skills and the acquisition of knowledge. Accurately simulating the sense of touch (inherent in psychomotor skills development) in the virtual domain is challenging, due to various technical limitations and cost issues. Pseudo-haptics refers to the simulation of haptic sensations using commonly available devices and equipment available to the typical computer user at their home (e.g., computer mouse). We do acknowledge limitations with pseudo-haptics, and more specifically, the ability to faithfully simulate the full range of psychomotor-skills without the use of haptic devices. Although greater work remains before more concrete statements can be made, we believe that incorporating pseudo-haptics within iVLEs can serve an important function and bridge the gap between the currently available iVLEs that focus solely on cognitive skills development, and those complex and costly iVLEs that do account for psychomotor skills development housed in research laboratories and are therefore limited in availability. More specifically, pseudo-haptics in simulations will allow trainees to practice various psychomotor skills-based tasks at home using standard computer hardware and equipment and although the fidelity and corresponding feedback will be limited, it can serve to better prepare trainees for when they move into the simulation laboratories and thus ensure that they better utilize their time in these laboratories.

Although our work has so far focused on surgical drilling, it is anticipated that these results will generalize to a variety of psychomotor-based tasks (e.g., suturing, and needle insertion). After further experimentation, a set of (preliminary) guidelines will be devised. These guidelines will guide designers and developers of healthcare-based iVLEs who wish to focus on technical skills development utilizing pseudo-haptics with common computer hardware devices.

## References

[1] Perkins GD (2007). Simulation in resuscitation training. Resuscitation.

[2] Lin Y, Cheng A, Hecker K, Grant V, Currie GR (2018). Implementing economic evaluation in simulation-based medical education: challenges and opportunities. Med Educ.

[3] Kapralos B, Uribe-Quevedo A, Dubrowski A (2017). Immersive technologies for medical education. Encyclopedia of Computer Graphics and Games.

[4] Zhou M, Tse S, Derevianko A, Jones DB, Schwaitzberg SD, Cao CG (2012). Effect of haptic feedback in laparoscopic surgery skill acquisition. Surg Endosc.

[5] Collins K, Kapralos B (2019). Pseudo-haptics: a review of leveraging cross-modal perception in virtual environments. Senses Soc.

[6] Aldridge R, Carr KT, England R, Meech JF, Solomonides T (1996). Getting a grasp on virtual reality. Int Conf Comput Hum Interfaces.

[7] Lécuyer A, Burkhardt J-M, Etienne L (2004). Feeling bumps and holes without a haptic interface. Proc SIGCHI Conf Hum Factor Comput Syst.

[8] Li M, Liu H, Li J (2012). Tissue stiffness simulation and abnormality localization using pseudo-haptic feedback. 2012 IEEE Int Conf Robot Autom.

[9] Ujitoko Y, Ban Y (2021). Survey of pseudo-haptics: Haptic feedback design and application proposals. IEEE Trans Haptics.

[10] Kinoshita H, Nagahata M, Takano N (2016). Development of a drilling simulator for dental implant surgery. J Dent Educ.

[11] Correa CG, Tokunaga DM, Ranzini E, Nunes FLS, Tori R (2016). Haptic interaction objective evaluation in needle insertion task simulation. Proc 31st ACM Symp Appl Comput.

[12] Wang Q, Qin J, Wang W, Shan J, Zhang J, Liu X, Heng P (2015). Haptic rendering of drilling process in orthopedic surgical simulation based on the volumetric object. Proc 2015 IEEE Int Conf Digit Signal Process Proc.

[13] Ning G, Daggett Q, Perivolaris A (2021). Rethinking audio-haptic perceptual immersion from in-person to remote testing during COVID-19. Proc Int Conf Interact Mob Commun Technol Learn.

